# CRTSC: Channel-Wise Recalibration and Texture-Structural Consistency Constraint for Anomaly Detection in Medical Chest Images

**DOI:** 10.3390/s25216722

**Published:** 2025-11-03

**Authors:** Mingfu Xiong, Chong Wang, Hao Cai, Aziz Alotaibi, Saeed Anwar, Abdul Khader Jilani Saudagar, Javier Del Ser, Khan Muhammad

**Affiliations:** 1 School of Computer Science and Artificial Intelligence, Wuhan Textile University, Wuhan 430200, China; xmf2013@whu.edu.cn (M.X.); wangc683283@163.com (C.W.); 2 Department of Computer Science, College of Computers and Information Technology, Taif University, Taif 21974, Saudi Arabia; azotaibi@tu.edu.sa; 3 Department of Computer Science and Software Engineering, The University of Western Australia, Perth, WA 6009, Australia; saeed.anwar@uwa.edu.au; 4 Information Systems Department, College of Computer and Information Sciences, Imam Mohammad Ibn Saud Islamic University (IMSIU), Riyadh 11432, Saudi Arabia; aksaudagar@imamu.edu.sa; 5 TECNALIA, Basque Research & Technology Alliance (BRTA), 48160 Derio, Spain; javier.delser@tecnalia.com; 6 Department of Mathematics, University of the Basque Country (UPV/EHU), 48940 Leioa, Spain; 7 Visual Analytics for Knowledge Laboratory (VIS2KNOW Lab), Department of Applied Artificial Intelligence, School of Convergence, College of Computing and Informatics, Sungkyunkwan University, Seoul 03063, Republic of Korea

**Keywords:** unsupervised anomaly detection, medical image chest analysis, sensor-based medical applications, channel calibration, anomaly structure

## Abstract

Unsupervised medical image anomaly detection, which does not need any labels, holds a pivotal role in early disease detection for advancing human intelligent health, and it is among the prominent research endeavors in the realm of biomedical image analysis. Existing deep model-based methods mainly focus on feature selection and interaction, ignoring the relative position and shape uncertainty of the anomalies themselves, which play an important guiding role in disease diagnosis, hampering performance. To address this issue, our study introduces a novel and effective framework, termed CRTSC, which integrates a channel-wise recalibration module (CRM) along with the texture–structural consistency constraint (TSCC) for anomaly detection in medical chest images acquired from different sensors. Specifically, the CRM adjusts the weight of different medical image feature channels, which are used to establish spatial relationships among anomalous patterns, enhancing the network’s representation and generalization capabilities. The texture–structural consistency constraint is devoted to enhancing the anomaly’s structural (shape) definiteness via evaluating the loss function of similarity between two images and optimizing the model. The two collaborate in an end-to-end fashion to optimize and train the entire framework, thereby enabling anomaly detection in medical chest images. Extensive experiments conducted on the public ZhangLab and CheXpert datasets demonstrate that our method achieves a significant performance improvement compared with the state-of-the-art methods, offering a robust and generalizable solution for sensor-based medical imaging applications.

## 1. Introduction

**Figure 1:**
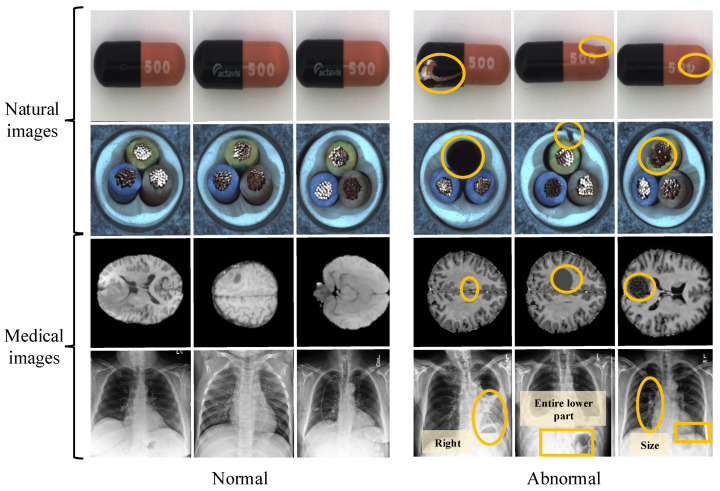
The first two rows represent natural images, and the yellow circles highlight multiple types of defects: sealing defects, dents, and scratches in the first row; hollow, insulation layer defects and conductor deviation in the second row. The third row contains normal and abnormal (inflammatory, lesions and bleeding) MRI medical images, and the fourth row shows the normal and abnormal (pneumonia, effusion, and nodule) medical images from the ZhangLab dataset. Unlike natural images, anomaly detection in smart health medical images focuses more on the individual shape and location of the target for accurate diagnosis.

As a significant research endeavor in the field of biomedical image analysis, unsupervised medical image anomaly detection (UMIAD) has attracted considerable attention in medical engineering due to its ability to efficiently identify abnormal patterns in medical scan images without the need for any data annotation [1,2]. Due to its data economy and friendliness (without any labels), it has become one of the current hot research topics in the intersection of medical and industrial fields, which is also a measure of the completeness of a country’s medical and health system [3,4,5]. Especially in clinical medical research, if medical image recognition is used to detect abnormal patterns in the human body in advance, it will facilitate the early detection of potential diseases and significantly shorten the treatment cycle [6,7]. Unlike natural images, object detection in medical images (e.g., X-ray, CT, or MRI) emphasizes the specific localization of targets, as illustrated in Figure 1. Their relative position and direction of target structures are essential focus points recognized in this task. Due to objective factors such as complex structure, noise interference, and data diversity, UMIAD remains a challenging task [3,8].

Recently, deep learning-based methods have made significant advances in image anomaly detection and actual application [9,10,11], especially in medical images, and they can be used in medicine for tumor detection, bone fracture identification, disease marker detection, and abnormal organ recognition [12,13]. Generally, traditional UMIAD-based methods are classified into three classes: (1) image reconstruction, (2) self-learning, and (3) pre-trained models-based. The first category is devoted to reconstructing the normal image samples by preventing low reconstruction errors, assuming they are short of abnormal ones. Although these methods have achieved satisfactory results, satisfying samples with subtle anomalies are not easy [14]. The second category has mainly explored contrastive learning, using pre-designed models to simulate normal and abnormal samples for detection. However, this strategy struggles to effectively adapt to new defect samples in the presence of data shortage or their limited representability of all possible anomaly types [15,16]. The last type of method involves pre-training the model in a source domain and testing it in the target domains [17,18]. Due to the differences in data distribution between the source and target domains, it is not easy to train a model with strong robustness.

Lately, the academic community has made some new progress in UMIAD, with representative works such as Thomas et al. [17] proposing a new framework for Patch Distribution Modeling to concurrently detect and localize anomalies in images within a one-class learning setting. Also, it exploits the correlations between different semantic levels of CNNs to aid in localization. Xiang et al. [3] proposed a SQUID method to minimize the structured information of abnormal targets to achieve medical image anomaly detection. Their method used a recursive manner to complete the fine-grained structural dissection inference model of abnormal targets to identify anomalies in medical images. Although existing methods have progressed, they mainly focus on feature selection and interaction, ignoring individual differences and hampering detection performance, which may lead the model to mistakenly identify certain areas of a normal image as anomalies. Moreover, the presence of noise can hinder reconstruction methods from effectively detecting and restoring anomalies, particularly those with blurred edges and intricate details. Specifically, medical image detection focuses more on the relative position of abnormal objects and their shape (size, dimension, etc.), shown in the fourth row of Figure 1. The yellow circles from rows one to four indicate the following anomalies, respectively: sealing defects, dents, and scratches in the first row; hollow and insulation layer defects such as conductor deviation in the second row; inflammatory signs, lesions, and bleeding in the third row; and pulmonary abnormalities including pneumonia, effusion, and nodules in the fourth row, which have positive guidance and reference significance for disease diagnosis. Therefore, if the above factors (relative position and shape uncertainty of the anomalies) are ignored, performance will inevitably decrease.

To overcome this problem, this study proposes a novel and effective CRTSC framework, coined as the channel-wise recalibration along with the texture–structural consistency constraint, to mine the individual features of abnormal samples for chest UMIAD. The proposed framework includes two components: (1) a channel-wise recalibration module (CRM) and (2) a texture–structural consistency constraint (TSCC). The former adjusts the attention of different medical image feature channels, enhancing the network’s representation capability. Specifically, it is used to establish and adapt spatial relationships channel-wise, which maintains the original dimension and the individual spatial location relationship, reducing the number of parameters and preventing the model from misjudging certain regions. In addition, the proposed TSCC loss calculates the similarity differences to enhance the sample’s structural differences. It combines brightness, contrast, texture, and structural information (e.g., contour) to preserve individual shapes’ details and improve the quality of generated images, effectively mitigating the influence of noise. Extensive experiments and ablation analysis demonstrate that our proposed method is superior to the latest UMIAD methods on public medical chest image datasets, including ZhangLab Chest X-ray [19] and Stanford CheXpert [20]. Particularly on the ZhangLab dataset, our method achieves a better accuracy than its benchmark and has the best F1 score.

In short, the main contributions of this work are summarized as follows.

- This study proposes a novel and effective framework called channel-wise recalibration along with the texture–structural consistency constraint (CRTSC), which includes a channel-wise recalibration module (CRM) and a texture–structural consistency constraint (TSCC) for unsupervised medical image anomaly detection.
- The proposed CRM and TSCC modules establish spatial relationships between abnormal objects and enhance their definiteness to preserve the uniqueness of individual samples, respectively.
- Extensive experiments and ablation studies have been conducted on the UMIAD public large-scale datasets, including the ZhangLab Chest X-ray and Stanford CheXpert, proving the promising results of our method against SOTA approaches.

## 2. Related Works

### 2.1. Anomaly Detection

This area aims to identify special instances or observations in a dataset that differ from most of the data, which are often called outliers or anomalous data [1]. It is often used in classification and object detection tasks [3,14]. Chandola et al. [1] reviewed anomaly detection based on the traditional methods, which include the support vector machine (SVM) [21], random forest [14], and other traditional machine learning methods for classification and detection. They are mainly divided into image reconstruction-based and recovery-based methods [22]. Reconstruction-based methods train a model on normal samples to recover the original split one and discriminate abnormalities by reducing the reconstruction error [23]. Image recovery-based methods consider anomalies to be noise that can be detected using the denoising strategy [24]. In addition, the deep autoencoder [25] and U-Net [26] are standard anomaly detection methods. However, the distribution of normal images learned by these methods does not account for potential anomalies and overlooks the interrelationships between convolutional channels. This paper introduces the channel-wise recalibration module to address these limitations. Recently, with the rise of deep learning, this branch of artificial intelligence has also been widely used in various anomaly detection applications [27,28]. For example, several previous works have investigated image inpainting for anomaly detection [6,29]. Abdelaty et al. [15] proposed a deep learning framework called “DAICS” with a two-branch structure to undertake anomaly detection in industrial applications.

### 2.2. Unsupervised Medical Image Anomaly Detection

Medical image anomaly detection can effectively discover the characteristics of diseased areas, providing timely auxiliary therapy for clinical diagnosis, and it has become a research area in the intersection of medicine and engineering [3,30]. For example, Yu et al. [4] proposed the MemMC-MAE method to mask the input samples and reduce the risk of producing low reconstruction error for UMIAD. Similarly, Marimont et al. [25] designed an auto-decoder network to model the distribution of normal images. Pavlovsky et al. [31] proposed a method with the Bayesian-based autoencoder model for regular data training that is used to detect anomalous objects for brain CT scan applications. However, their methods are mainly suitable for regular images and may exhibit poor detection performance for certain atypical anomalies. Although Xiong et al. [3] and Zhao et al. [32] achieved commendable experimental results in UMIAD, they overlooked the importance of feature channels, making it difficult to solve the misjudgment caused by the uncertainty of abnormal shapes. Zhou et al. [33] proposed a novel Proxy Bridged Image Reconstruction Network (ProxyAno) for anomaly detection in medical images, using an intermediate proxy to bridge the input and reconstructed images. Other unsupervised methods include generative adversarial networks [34] and GANomaly [35,36]. Unlike existing methods, our approach focuses primarily on the relative position of abnormal targets, as well as their shape, size, and scale features in medical images, aiming to mitigate misjudgments caused by noise interference and uncertainty in abnormal shapes within the model.

## 3. Proposed CRTSC Method

**Figure 2:**
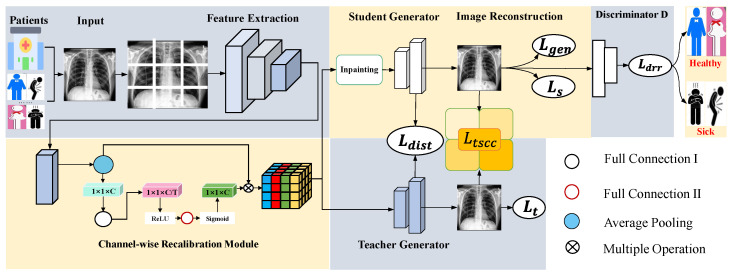
Proposed CRTSC framework, which includes the feature extraction part, the channel-wise recalibration module (Section 3.1), and the texture–structural consistency constraint loss module (Section 3.2). We divide an input image into N×N non-overlapping patches. Two generators are trained to reconstruct the original image. Finally, a discriminator is used to determine whether the reconstructed patient radiography image is healthy or sick.

In this section, we present the complete framework of our method, which is illustrated in Figure 2. The following sections introduce our proposed modules (CRM in Section 3.1 and TSCC in Section 3.2) and anomaly discrimination (Section 3.3). Finally, Section 3.4 provides details about our method’s loss function and training process.

### 3.1. Channel-Wise Recalibration Module

Various challenges, such as feature expression, selection, dimension transformation, and inconsistency, arise frequently when extracting features. Inspired by [3], our proposed CRTSC framework embeds a channel-wise recalibration module (CRM) that simulates the interdependence between convolutional channels, enabling the network to perform feature attention, highlight essential information, suppress irrelevant content, and improve the network’s representation ability.

The specific execution process of the CRM is as follows: First, the input image is divided into N×N patches. Since different datasets contain images of varying resolutions, the value of N is determined based on the original input size to ensure complete and non-overlapping coverage (2 in our case) and fed into an encoder (Transformer [37] as the backbone) for feature extraction, which is used for image reconstruction after subsequent operations. In this study, we utilized the most primitive convolutional layer, the pooling layer, and BatchNorm2d. It comprises two sequential layers. The first one performs a convolution, followed by normalization and activation of the output. The second one includes two consecutive convolution operations, with activation in between, and batch normalization after each convolution, aiming at deeper feature extraction. To preserve spatial and channel-wise recalibration features, we adopt the CRM mechanism, which processes spatial and channel information separately. The CRM establishes relationships among different channels and assigns weights to them. It does not change the original spatial dimensions but helps maintain spatial information, reduce the number of parameters, and assist the model in reducing misjudgments when the relative position and shape of the anomaly are uncertain.

The training details of this module encompass forward propagation, backward propagation, and parameter updating. During forward propagation, the H × W spatial information from each channel is compressed into a single scalar value. This compression acts as a strong structural prior, enabling each channel descriptor to maintain a global receptive field over the entire feature map, regardless of its original spatial dimensions. As a result, the CRM exhibits enhanced robustness to spatial transformations (including variations in target position and scale) within the input image. Subsequently, a fully connected layer combined with activation functions is employed to perform dimensionality reduction, linear transformation, dimensional expansion, and weight restriction. Finally, the learned weight vector is applied to the original feature map through channel-wise multiplication, producing the final output. During backpropagation, the gradient of the loss function flows simultaneously into two pathways (the original feature branch and the weight calibration branch) via the weighting operation. The gradient propagates through both the fully connected layer and the activation function before reaching the initial compressed statistic $z_{c}$ with parameter updates across the two fully connected layers being computed using the chain rule.

Dimension reduction can enhance the generalization ability of the network, lower computational costs, and avoid the risk of overfitting, thus improving the efficiency of the model. Subsequent reweighting strategies are employed to restore the original feature dimensions (In Equation (4)) and optimize the model by adjusting the dimensionality reduction ratio, which is a practical choice in UMIAD [4]. The feature map transformation performed within the CRM mathematically is denoted as Equation (1):

(1) $$X⊛M,\;\mathrm{with}\;X\in R^{W^{\prime }\times H^{\prime }\times C^{\prime }},M\in R^{W\times H\times C},$$

where *X* and *M* represent the features before and after transformation, respectively; ⊛ is a standard convolution operation; and $W\;(W^{\prime })$, $H\;(H^{\prime })$, and $C\;(C^{\prime })$ denote the weight, height, and channels of convolutional features, respectively.

For the converted input feature map, the global average pooling operation is used to compress each channel, which reduces the dimensionality of feature tensors and transforms them from three-dimensional to one-dimensional. The purpose of this step is to capture the global statistical information of each channel, adjust the feature weights based on the average feature values of each channel, and make the subsequent learning process more efficient. After that, we have a feature tensor of size 1×1×C (where *C* represents the number of channels). Especially in our experiments, the proportion parameter for channel reduction is set to a large number, significantly reducing the dimensionality and computational complexity and making the experimental effects more prominent. However, it would result in information loss in the compression processes for each channel. The feature enhancement and aggregation will offset this loss later. The formula is given by Equation (2):

(2) $$z_{c}=\frac{1}{H\cdot W}\sum_{i=1}^{H}\sum_{j=1}^{W}m_{c}\left(i,j\right),$$

where $m_{c}$ denotes the *c*-th channel of the input feature map and $z_{c}$ is the output of the corresponding channel *c*, i.e., the specific value after global average pooling.

During the channel compression process in the spatial dimension, valuable information is consolidated. Since a single fully connected layer cannot effectively accommodate the ReLU and sigmoid nonlinear functions, we adopt a strategy to feed feature vectors to two separate fully connected layers. This allows the model to learn the weights associated with each channel, enabling it to discern the importance of each channel and to achieve an enhanced focus on critical features.

Different channel weights signify distinct channels of information. Since the dimension reduction ratio influences the model’s performance, it is crucial to determine an optimal value. Through tuning and validation experiments conducted after model training, we found that setting the ratio to 24 (as elaborated in the subsequent experimental section) yields improved experimental outcomes. Increasing this ratio reduces computational complexity and the number of parameters, thus enhancing the model’s training efficiency and effectiveness. The vector *z* passes through the first fully connected layer with the dimension changing from 1×1×C to 1×1×C/T and then through the second fully connected layer with the dimension changing from 1×1×C/T to 1×1×C, where *T* is a constant representing an integer with reduced dimensionality. One is a descending layer, and one is an ascending layer, utilizing the ReLU and sigmoid functions, respectively. We also use a gating mechanism to obtain channel dependencies and activate them with the sigmoid function. The recalibrated weights are applied to each channel on the original feature map, multiplying each channel’s features by the corresponding weights. According to different values, the module adjusts the importance of the channel to obtain the final feature map as Equations (3) and (4):

(3) $$s=\sigma \left(fc_{2}\left(ReLu\left(fc_{1}z\right)\right)\right),$$

(4) $$M_{c}=F\cdot s,$$

where $M_{c}$ is the attention map, *s* is the weight obtained by activating the sigmoid function, *F* is the input feature map, σ(·) represents the sigmoid function, ReLU is the nonlinear activation, and $fc_{1}$ and $fc_{2}$ denote the weight matrices of the two fully-connected layers, which process the vector *z* to obtain the channel weight value.

### 3.2. Texture–Structural Consistency Constraint

The structural similarity index is a metric used to gauge the similarity between two images, serving as a yardstick for assessing image quality. The TSCC function, designed based on this metric, is integrated into CRTSC to alleviate the impact of noise, which complicates the model’s ability to accurately capture and restore anomalies with complex details and blurred edges during the reconstruction process, often overlooking individual structural differences between generated and original images.

Unlike traditional approaches that solely calculate image differences, TSCC not only takes into account factors such as changes in luminance, contrast, and structural information but also considers the influence of texture similarity. By capturing high-frequency information, such as edges, details, and complex patterns, TSCC offers a more comprehensive quality assessment, significantly enhancing the accuracy and reliability of detection. This approach helps preserve or restore details during image processing, aiding in the reconstruction of fine details and preventing excessive smoothing and blurring. In this paper, we employ a texture consistency calculation method based on local binary pattern (LBP) [38], a technique that compares each pixel in an image with its neighboring pixels to generate binary patterns. This method effectively captures the texture features of the image. In the generation of LBP, the number of neighborhood sampling points and the radius of the circular neighborhood are controlled by parameters *P* and *R*, respectively. In this study, we set P = 8 and R = 1, corresponding to a neighborhood patch of size 3×3. We adopt the “uniform” LBP formulation, which yields a total of 59 distinct texture features: 58 uniform binary patterns and 1 composite bin for all non-uniform patterns. By incorporating TSCC into the reconstruction losses, our CRTSC can better preserve image details and structural information, thereby enhancing the quality of generated images. During training, TSCC optimizes the image quality produced by the student generator, guiding the model to generate more realistic images. This module plays a crucial role in refining the reconstruction process and improving the overall performance of the CRTSC model.

Luminance, contrast, and structural features refer to the respective similarities between the reconstructed image and the input image. The formula is denoted as Equations (5)–(7):

(5) $$l\left(r,i\right)=\frac{2Mev\left(r,i\right)+K_{1}}{Mev^{2}\left(r\right)+Mev^{2}\left(i\right)+K_{1}},$$

(6) $$\mathrm{cs}\left(r,i\right)=\frac{2Cov\left(r,i\right)+K_{2}}{Cov^{2}\left(r\right)+Cov^{2}\left(i\right)+K_{2}},$$

(7) lcs(r,i)=1−l(r,i)·c(r,i),

where *i* and *r* represent the original and reconstructed images; Cov(r,i) is covariance and Mev(r,i) stands for their mean values of luminance; $K_{1}$ and $K_{2}$ are constants added for stability, which have a small value, and we set them to $0.01^{2}$ and $0.03^{2}$ respectively; and lcs(r,i) is the comprehensive loss function that integrates brightness, contrast, and structural similarity.

The texture similarity measurement of texture information between input and reconstructed images is described as Equation (8):

(8) $$t\left(r,i\right)=\frac{1}{N}\sum_{j=1}^{N}\left|L{\left(r\right)}_{j}-L{\left(i\right)}_{j}\right|,$$

where *N* represents the total number of texture features; $L{\left(i\right)}_{j}$ and $L{\left(r\right)}_{j}$ represent the LBP values of the original *i* and reconstructed images *r* at position *j*, respectively.

The similarity range for each type is from 0 to 1. The closer the similarity value is to 1, the higher the similarity between the two images in that aspect. The final TSCC function $L_{tscc}$ is given by (9):

(9) $$L_{tscc}\left(r,i\right)=lcs\left(r,i\right)+t\left(r,i\right),$$

Traditional SSIM losses [39] mainly focus on the traits of human eye perception to determine the similarity between the images, namely, the impact of brightness and contrast. Our proposed TSCC ($L_{tscc}$) function incorporates texture similarity into the original loss function, emphasizing the structural consistency among individual samples, which is more applicable in medical anomaly detection.

### 3.3. Anomaly Discrimination

The discriminator plays a crucial role in anomaly detection by assessing the similarity between the reconstructed image and real data. Therefore, after implementing the CRM and TSCC modules, we can determine the location and shape of the abnormal target. Due to the error between the reconstructed image and the real one, we need to reduce their errors through anomaly discrimination. We have proposed two generators (teacher and student ones) to discern the discrepancies between real and generated data, as illustrated in Figure 2. Specifically, the student generator generates normal images, while the teacher generator adjusts it to produce diverse images. The disparities between input and generated images decrease under normal conditions as training progresses. This study employs the SimpleDiscriminator [40] for anomaly detection due to its fewer parameters and faster training speed. The so-called anomaly score A (an anomaly metric indicator [3]) quantifies the resemblance between input data and real data, with higher scores indicating a higher likelihood of the input image being an anomaly and lower scores suggesting a higher likelihood of the input image being realistic.

### 3.4. Framework Optimization and Loss Function

From the baseline in [3] and our proposed method, we have six loss functions. The other five are the mean square error $L_{t}$ and $L_{s}$ [3] of the teacher and student generators concerning the input image, the distance constraint error $L_{dist}$ [3], the generation error $L_{gen}$ [34], and the discriminator error $L_{drr}$ [3]. The teacher and student generators follow the knowledge distillation paradigm. When chest anomalies are not apparent, cross-entropy loss may be unable to handle uncertainty, leading to poor training of the model. So, the generator loss is changed to the Kullback–Leibler (KL) [41] scatter loss function. This change aims to help the model discern the distinction between the target and generation distributions, thereby enhancing the disparity between the model-generated and original images. Here, the KL scatter and adversarial loss functions (like DCGAN [34]) are used together to train the generator. Therefore, the total loss function is given by (10):

(10) $$\ell =\lambda_{t}L_{t}+\lambda_{s}L_{s}+\lambda_{tscc}L_{tscc}+\lambda_{dist}L_{dist}+\lambda_{gen}L_{gen},$$

where λ denotes the hyperparameter of each loss function. CRTSC is trained to minimize the total loss function and maximize the discriminator loss $\lambda_{drr}L_{drr}$.

## 4. Experimental Results and Analysis

In this section, we provide the experimental results and their analysis. Firstly, the experimental setup is described in Section 4.1. Then, the comparison with state-of-the-art (SOTA) methods is described in Section 4.2. Finally, results of an ablation study are presented and discussed in Section 4.3. The details are described as follows.

### 4.1. Experimental Setup

Datasets Description: In our experiments, we utilize the ZhangLab Chest X-ray [19] dataset, abbreviated as ZhangLab; and Stanford CheXpert [20], termed as CheXpert dataset. Both are publicly available. The ZhangLab dataset comprises normal and anomaly images, with pneumonia representing the anomaly class and others representing healthy chest images. The dataset includes train and test sets, totaling 4856 images. The training set consists of 4232 images with 1349 normal and 3883 anomaly images, while the test set contains 624 images, in which 234 are normal and the rest are abnormal. All images are resized to 128×128 for the experiments. The CheXpert dataset comprises 23,671 anomalies and 5249 normal images, while 250 anomalies and 250 normal images are reserved for testing.

Implementation Details: We employed PyTorch (version 1.12.1+cu113) to implement CRTSC and the conducted experiments, running the model on an NVIDIA RTX2080 Ti GPU. Our CRTSC model begins with a feature extractor responsible for feature extraction, comprising four convolutional layers. In the proposed CRM module, we initially downsample the features using a pooling layer to reduce the feature map’s size. Subsequently, two fully connected layers are employed for feature recalibration. In the final discriminator, five convolutional layers are utilized to perform convolution operations on the image generated by the generator, resizing it from the size of the generated image 1 × 128 × 128 to 128 × 4 × 4. The image is processed through a fully connected layer to obtain the final image (1 × 1 × 1).

Parameters Setting: Adam [42] is used as the solver with a batch size of 16, a weight decay of $1\times 10^{-5}$, and an initial learning rate of $1\times 10^{-4}$. We use a learning rate scheduler, setting the epochs to 800 on the ZhangLab dataset and the minimum learning rate to 0.1 times the initial learning rate. Training is conducted for 600 epochs on the CheXpert dataset. The weight parameters of the loss function are set as: $\lambda_{t}$ = 0.01, $\lambda_{s}$ = 10, $\lambda_{dist}$ = 0.001, $\lambda_{gen}$ = 0.002, and $\lambda_{drr}$ = 0.005. Meanwhile, $K_{1}$ and $K_{2}$ are set as $0.01^{2}$ and $0.03^{2}$, respectively. For the input, the medical images of size 128 × 128 are divided into 2 × 2 subimages of size 64 × 64.

Evaluation Metrics: We assess the performance of our model in terms of the receiver operating characteristic (ROC) curve, area under the ROC curve (AUC), accuracy (ACC), and F1-score (F1) [19].

### 4.2. Comparisons with State-of-the-Art Methods

We first evaluate our CRTSC framework and compare its performance with that of several recently published techniques that have considered these datasets, including SALAD [32], IF-2D [25], IGD [21], M-KA [43], PANDA [6], SQUID [3], and SimSID [30], among others. All comparison data are based on baseline results. We compare the final training results of CRTSC with the baseline results on the ZhangLab dataset. The experiments for CRTSC on both datasets are conducted using the same equipment and experimental conditions. Table 1 and Table 2 summarize the obtained performance results.

**Figure 3:**
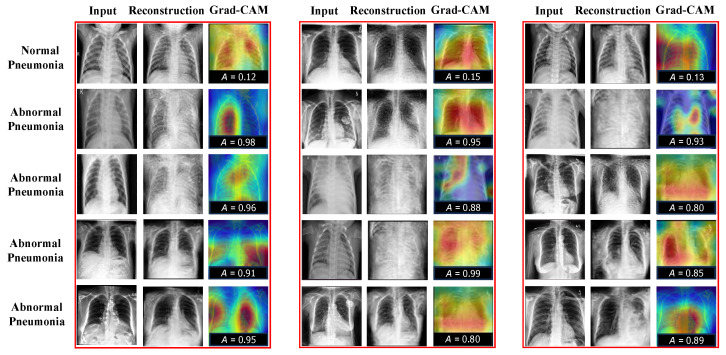
Reconstruction results on the ZhangLab and CheXpert datasets. “A” denotes the structural similarity. The higher this score is, the greater the probability of being abnormal will be. “Reconst” denotes the reconstruction images. “Grad-CAM” refers to gradient-weighted class activation mapping, used to visualize the parts of importance of image regions for the classification made by the model (healthy vs sick). Grad-CAM (Gradient-weighted Class Activation Mapping) is used to visualize which regions of an image contribute most to a model’s classification decision (In our case, *healthy* vs. *sick*). Warm colors such as red, orange, and yellow highlight areas that strongly influence the prediction toward the *sick* category, indicating potential anomalies. Conversely, cooler colors like blue and green correspond to regions associated with healthy tissue, pushing the decision away from the *sick* category towards the *healthy* label.

As shown in the above tables, our CRTSC framework yields notably superior accuracy scores, particularly on the ZhangLab dataset, where CRTSC outperforms all comparison baselines in the three performance scores considered. Specifically, CRTSC attains gaps of 1% (AUC), 3% (ACC), and 3% (F1) concerning the best condition in the benchmark. Results on the CheXpert dataset expose a significant improvement over the baseline method. In Figure 3, we illustrate the reconstruction results of normal and abnormal images by CRTSC on the ZhangLab dataset, alongside their corresponding anomaly scores. As can be observed in this figure, the reconstructed image by the CRTSC framework closely resembles the input image when it is not an anomaly, which is further validated by the Grad-CAM heatmaps displayed for every case.

### 4.3. Ablation Studies

In this part, we evaluate the effectiveness of the proposed single module and loss functions in the CRTSC framework. It is performed via a comprehensive ablation study on the ZhangLab and CheXpert datasets, which includes the analysis of components, KL divergence weights, CRM parameters, and TSCC weights, respectively. The details are described as follows.

Components Analysis: We conduct ablations by integrating the proposed single component (KL loss, TSCC, and CRM) into the baseline, which is shown in Table 3. We observe that the different components can contribute differently to the performance, with CRM achieving the largest effect. The bold formatting indicates the best performance achieved across all configurations for each individual metric column, which may not represent the overall optimal performance of the complete CRTSC model. The final row of the table (our complete model) presents the integrated performance obtained by combining all three proposed modules. These results are not highlighted in bold as they do not surpass the top results achieved by any single-module configuration in any metric column, which is consistent with the established formatting convention. Although the F1-score remains marginally below the baseline level, both the AUC and ACC metrics demonstrate a clear improvement over the baseline. Specifically, we replace the loss function of the generator in the baseline with KL loss, which significantly enhances the model accuracy. Table 3 presents the specific ablation results, indicating a notable improvement in ACC and F1 metrics by adding the KL loss function to the baseline. Notably, the CRM and TSCC modules emerged as the most influential contributors. Consequently, the final results of our CRTSC surpass those of the baseline.

KL Weights Analysis: We also conduct experiments for different weights of KL loss functions on the ZhangLab and CheXpert datasets, which is shown in Table 4. For the KL scatter loss function, the weight of the cross-entropy loss function in the baseline is initially set to 0.005. Through extensive experiments, we determined that the best results are achieved when the KL weight is set to 0.002. The result of the first row (baseline) is $\lambda_{gen}$ = 0.005. This adjustment was made solely by replacing the cross-entropy loss with the KL loss function in the baseline. We found out that the weight values were effective for the model and maintained it at 0.002 even after the final integration of the CRM and TSCC modules.

CRM Parameters Analysis: As mentioned above, adding the CRM module would enhance the feature representation ability of the network. To confirm this statement, we also conducted a series of experiments on the ZhangLab and CheXpert datasets, which are shown in Table 5. It can be seen that the evaluation metrics AUC (88.98%), ACC (82.53%), and F1 (86.29%) show significant improvement, making it a valuable addition to the model. Subsequently, after incorporating the TSCC loss function, we found that setting its reduction value to 24 yields the best overall performance, enhances efficiency, and reduces computational complexity. This configuration leads to a substantial improvement across all three metrics. In particular, we have exploited different reduction values (8, 12, 16, 20, and 24) to verify the final results. It can be seen that the model achieved the highest effectiveness when the reduction value was set as 8 and 16 on the ZhangLab and CheXpert datasets, respectively.

**Figure 4:**
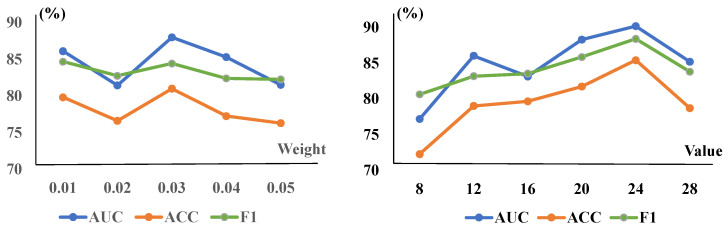
The CRM module’s parameters and the TSCC module’s weights $\lambda_{tscc}$ are trained on the ZhangLab dataset. The (**left**) graph is for the weight of TSCC, while the (**right**) one shows the reduction of the CRM. The AUC peaked at a weight of 0.03 and a reduction value of 24 in the overall model, incorporating both modules.

TSCC Weights Analysis: The TSCC function measures structural similarity between the original and reconstructed images in this work. We also present corresponding quantitative experiments on the ZhangLab and CheXpert datasets for different $\lambda_{tscc}$, shown in Table 6. When the TSCC loss function is added to the baseline alone, the evaluation metrics—AUC, ACC, and F1—have improved to 87.93%, 80.99%, and 84.67% in the ZhangLab dataset, and 85.78%, 78.60%, and 77.57% in the CheXpert dataset, respectively, demonstrating the promising accuracy of this function in detecting image anomalies. Furthermore, we illustrate the impact of CRM and TSCC parameters on the overall CRTSC model. With the single-variable principle, we have compared different values of existing parameters while selecting the best among other parameters, as evidenced by Figure 4.

Computational Complexity: To demonstrate the efficiency of our algorithm, we have conducted a series of experiments to compare its performance against the baseline method (SQUID [3]) and SimSID [30]. In particular, the computational complexity of our proposed approach adheres to an O(N) time complexity, where *N* represents the number of elements in the input tensor *X*. Our model’s running time, model size, and parameters are described in Table 7.

**Figure 5:**
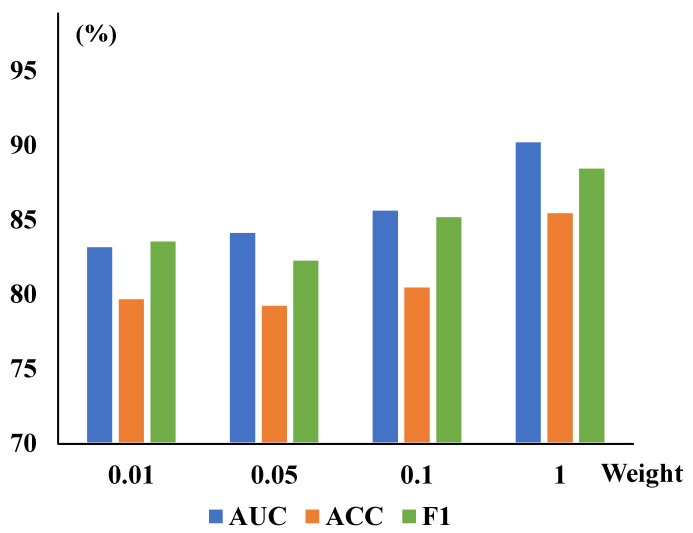
Different reduction and weight values are explored to optimize the CRTSC model. When the best optimal reduction is set as 24, different TSCC weight values are explored to optimize the model. It achieved the highest accuracy when the weight was 1.

In addition, to further demonstrate the performance of different hyperparameters of the TSCC and CRM modules, we have also conducted experiments to assess the impact of each parameter of the CRM module when TSCC parameters are optimized and vice versa. These are performed in the final comprehensive experiments, highlighting each module’s better robustness and optimal experimental scenarios. The tests are conducted on the ZhangLab datasets, and the specific results are presented in Figure 5. It can be seen that the value of AUC is highest when both values are optimal. In particular, to visually demonstrate the effectiveness of the algorithm proposed in this study, we have visualized the result, which is shown in Figure 3 with the reconstructed results on the ZhangLab and CheXpert datasets. The higher the score, the greater the probability of being abnormal.

## 5. Conclusions and Future Work

This study has introduced an unsupervised anomaly detection framework for biomedical imaging, termed CRTSC, which integrates two novel components: the channel-wise recalibration module (CRM) and the texture–structural consistency constraint (TSCC). The CRM enhances the model’s ability to capture and emphasize informative feature channels by dynamically adjusting their importance, thereby improving the network’s representational power. Complementarily, the TSCC module reinforces the structural fidelity of reconstructed images by incorporating luminance, contrast, and texture-based similarity metrics, which are particularly critical for detecting subtle anomalies in medical images.

Extensive experiments conducted on two publicly available chest X-ray datasets (ZhangLab and CheXpert) have demonstrated that CRTSC achieves competitive or superior performance compared with state-of-the-art unsupervised medical image anomaly detection methods. The results validate the effectiveness of our proposed modules in improving detection accuracy, robustness, and interpretability.

However, we acknowledge that the current evaluation is limited to chest X-ray modalities. While the ZhangLab and CheXpert datasets provide a solid foundation for validating our proposed approach, they do not encompass the full spectrum of medical imaging modalities such as CT, MRI, or ultrasound, each of which presents distinct structural, textural, and dimensional characteristics. Moreover, the performance variation observed between the two datasets (particularly the more heterogeneous and large-scale CheXpert dataset) highlights the challenges of generalizing across diverse clinical data sources.

To address these limitations, future work will focus on extending the CRTSC framework to a broader range of medical imaging modalities. This includes adapting the model to handle 3D volumetric data (e.g., CT and MRI), exploring modality-specific recalibration strategies, and integrating transfer learning and spatial pyramid inference mechanisms to enhance the localization and detection of anomalies across varied clinical scenarios. Additionally, we plan to investigate adaptive module integration and dataset-aware optimization techniques to improve robustness in the presence of domain shifts. Evaluating CRTSC on multi-institutional and multi-modal datasets will be a key step toward assessing and enhancing its generalizability and applicability in real-world healthcare environments.

## Figures and Tables

**Table 1:** Comparison of our CRTSC framework with state-of-the-art methods on the ZhangLab dataset based on AUC, accuracy, and F1-score. Best results for each score are highlighted in **bold**.

Models	Ref’Year	AUC (%)	ACC (%)	F1 (%)
MemAE [44]	ICCV’19	77.8 ± 1.4	56.5 ± 1.1	82.6 ± 0.9
MNAD [45]	CVPR’20	77.3 ± 0.9	73.6 ± 0.7	79.3 ± 1.1
SALAD [32]	TMI’21	82.7 ± 0.8	75.9 ± 0.9	82.1 ± 0.3
CutPaste [29]	CVPR’21	73.6 ± 3.9	64.0 ± 6.5	72.3 ± 8.9
PANDA [6]	CVPR’21	65.7 ± 1.3	65.4 ± 1.9	66.3 ± 1.2
M-KD [43]	CVPR’21	74.1 ± 2.6	69.1 ± 0.2	62.3 ± 8.4
IF-2D [25]	MICCAI’21	81.0 ± 2.8	76.4 ± 0.2	82.2 ± 2.7
PaDiM [17]	ICPR’21	71.4 ± 3.4	72.9 ± 2.4	80.7 ± 1.2
IGD [21]	AAAI’22	73.4 ± 1.9	74.0 ± 2.2	80.9 ± 1.3
SQUID [3]	CVPR’23	87.6 ± 1.5	80.3 ± 1.3	84.7 ± 0.8
SimSID [30]	TPAMI’24	**91.1 ± 0.9**	85.0 ± 1.0	88.0 ± 1.1
KD-MFAD [46]	SR’25	89.4 ± 1.5	**87.2 ± 1.3**	86.1 ± 1.1
CRTSC	Ours	88.7 ± 1.5	85.4 ± 1.7	**88.9 ± 0.6**

**Table 2:** Comparison of our CRTSC framework with state-of-the-art methods on the CheXpert dataset based on AUC, accuracy, and F1-score. Best results for each score are highlighted in **bold**.

Models	Ref’Year	AUC (%)	ACC (%)	F1 (%)
Ganomaly [35]	ACCV’18	68.9 ± 1.4	65.7 ± 0.2	65.1 ± 1.9
f-AnoGAN [36]	MIA’19	65.8 ± 3.3	63.7 ± 1.8	59.4 ± 3.8
MemAE [44]	ICCV’19	54.3 ± 4.0	55.6 ± 1.4	53.3 ± 7.0
CutPaste [29]	CVPR’21	65.5 ± 2.2	62.7 ± 2.0	60.3 ± 4.6
PANDA [6]	CVPR’21	68.6 ± 0.9	66.4 ± 2.8	65.3 ± 1.5
M-KD [43]	CVPR’21	69.8 ± 1.6	66.0 ± 2.5	63.6 ± 5.7
SQUID [3]	CVPR’23	78.1 ± 5.1	71.9 ± 3.8	**75.9 ± 5.7**
SSL [47]	CVPR’24	**80.3 ± 1.2**	56.2 ± 1.7	52.9 ± 0.7
KD-MFAD [46]	SR’25	74.0 ± 3.9	69.6 ± 3.0	70.9 ± 4.1
CRTSC	Ours	78.3 ± 0.6	**72.6 ± 1.2**	70.2 ± 2.3

**Table 3:** AUC, ACC, and F1 values for the KL, TSCC, and CWR modules are individually added to the baseline and integrated into the CRTSC model. Bold values shaded in blue highlight the best performance in each column.

Method	ZhangLab				CheXpert		
	AUC (%)	ACC (%)	F1 (%)		AUC (%)	ACC (%)	F1 (%)
w/o KL loss	87.66	82.53	86.59		78.21	71.90	75.97
w/o TSCC	87.99	81.41	85.71		79.03	72.61	**78.42**
w/o CWR	88.98	82.53	86.29		**79.65**	**74.00**	73.25
Baseline	87.60	80.30	84.70		78.10	71.90	75.90
CRTSC	**90.19**	**85.42**	**88.91**		78.26	72.60	70.20

**Table 4:** Results on different weights of the KL loss function. After extensive experiments, the KL weight is set to 0.002, achieving the best results on the ZhangLab dataset and 0.001 on the CheXpert dataset for AUC accuracy. Bold values shaded in blue highlight the best performance in each column.

$\lambda_{\mathrm{gen}}$	ZhangLab				CheXpert		
	AUC (%)	ACC (%)	F1 (%)		AUC (%)	ACC (%)	F1 (%)
Baseline	87.60	80.30	84.70		78.10	**71.90**	**75.90**
0.001	84.54	79.65	84.34		**78.26**	70.60	66.97
0.002	**87.66**	**82.53**	**86.59**		70.95	68.00	65.96
0.003	83.10	76.44	81.08		64.94	61.80	62.77

**Table 5:** Results on different reductions of the CRM module on the ZhangLab and CheXpert datasets. When adding the CRM module to the baseline solely, the reduction value of 8 yields the optimal results on the ZhangLab dataset, while the value of 16 achieves the best performance on the CheXpert dataset. Bold values shaded in blue highlight the best performance in each column.

Reduction	ZhangLab				CheXpert		
	AUC (%)	ACC (%)	F1 (%)		AUC (%)	ACC (%)	F1 (%)
24	79.73	74.04	79.70		69.26	65.40	66.79
20	86.03	79.01	83.18		67.39	65.60	60.73
16	79.73	82.53	**86.29**		**76.04**	**72.50**	**68.97**
12	84.01	77.40	82.22		69.55	66.20	65.01
8	**88.98**	**82.53**	**86.29**		70.47	65.60	66.27

**Table 6:** Results on different weights of the TSCC module on the ZhangLab and CheXpert datasets. Overall, the best performance can be achieved on both datasets when the $\lambda_{tscc}$ value is set to 0.03 and 0.02. Bold values shaded in blue highlight the best performance in each column.

$\lambda_{\mathrm{tscc}}$	ZhangLab				CheXpert		
	AUC (%)	ACC (%)	F1 (%)		AUC (%)	ACC (%)	F1 (%)
0.01	86.11	79.81	**84.67**		72.62	68.60	71.67
0.02	81.44	76.60	82.74		**85.78**	**78.60**	**77.57**
0.03	**87.93**	**80.99**	84.41		75.96	70.05	68.21
0.04	85.29	77.24	82.38		70.35	67.16	72.57
0.05	81.51	76.28	82.25		76.59	70.60	66.97

**Table 7:** Computational complexity of our CRTSC model. “Instances per Sec” denotes the number of instances the model recognizes per second.

Models	Parameters (M)	Flops (G)	Instances per Sec
SQUID [3]	44.47	7.6	260
SimSID [30]	82.24	7.6	216
CRTSC (Ours)	44.46	7.6	260

## Data Availability

The code and data are publicly available in the following repository: https://github.com/WANG683s/CRTSC, accessed on 22 October 2025.
